# Association between Physical Activity and Age among Children with Overweight and Obesity: Evidence from the 2016-2017 National Survey of Children's Health

**DOI:** 10.1155/2020/9259742

**Published:** 2020-09-24

**Authors:** Xun Li, Shi-Ting Xiang, Jie Dong, Yan Zhong, Sha Zhao, Zhenghui Xiao, Liping Li

**Affiliations:** ^1^Pediatrics Research Institute of Hunan Province, Hunan Children's Hospital, 86 Ziyuan Road, Changsha, China 410007; ^2^Institute of Child Health, Hunan Children's Hospital, 86 Ziyuan Road, Changsha, China 410007; ^3^Hunan Province Key Lab of Pediatric Emergency Medicine, Hunan Children's Hospital, 86 Ziyuan Road, Changsha, China 410007

## Abstract

Physical activity participation in children declines with age. It is not clear yet whether the age-related trends vary by weight status. This study is aimed at investigating the association between physical activity participation and age among children with healthy weight, overweight, or obesity, using data from the 2016-2017 National Survey of Children's Health (NSCH). Physical activity participation was evaluated by days participated in physical activity for at least 60 minutes out of 7 days. Weight status was categorized from body mass index (BMI) percentiles. Data were analyzed on 33,056 US children age 10-17 years. The percentages of been active 0 day out of 7 days in BMI5th < 85th (healthy weight), 85th < 95th (overweight), and ≥95th percentile (obese) groups were 8.9%, 11.5%, and 18.2%, respectively. Among all groups, been active 0 day out of 7 days was positively associated with age, while the strongest associations were observed in the BMI85th < 95th group (age 17 years vs. age 10 years: OR = 7.48, *p* < 0.0001). Older age was significantly associated with been active less than 4 days out of 7 days in the BMI5th < 85th and 85th < 95th groups, but those associations were attenuated in the BMI ≥ 95th group. This study found that physical activity participation was inversely associated with age among children with healthy weight, overweight, or obese, and the association was strongest among children with overweight and weakest among children with obesity. Interventions aimed at promoting physical activity among children should take these patterns of association into account.

## 1. Introduction

Maintaining physical activity participation throughout childhood is important for promoting lifelong health [1–4]. Based on the best available evidence, several global and national guidelines recommend that children and adolescents should accumulate a minimum of 60 minutes of moderate-to-vigorous physical activity daily [5–8]. However, survey data from many countries showed that a significant proportion of children and adolescents did not meet this recommendation [9, 10]. For example, the reported rates for children that met the recommendation in US and China were 26.1% and 34.1%, respectively [9, 11].

An important feature of physical activity participation in children and adolescents is that it declines with age [12, 13]. Childhood and adolescence are transitional periods of life marked by many biological, environmental, social, and psychological transformations, which influence changes in physical activity [14, 15]. The age-related decline of physical activity is a contributory factor to the development and maintenance of overweight and obesity [16, 17]. Results from a cohort study showed that, compared to consistently active participants, participants who were active as children but decreased activity with age were more likely to be affected by obesity [16]. And becoming inactive during the transition from adolescence to adulthood is associated with higher risk of obesity [17].

Although substantial public health efforts have been made to promote physical activity and to prevent age-related decline, interventions so far have limited success [18, 19]. From a population health perspective, identifying the patterns of change in physical activity participation is critical for the development of intervention programs. The changes of physical activity participation in children with overweight or obesity might be different from their healthy weight contemporaries, as overweight and obesity could reinforce the inactivity [20, 21]. However, previous studies that investigated the association between physical activity participation and age did not subgroup by weight status, and the associations in each subpopulation are not clear yet.

Given the current gaps in the literature, we conducted the present study to investigate the associations between physical activity participation and age among children with healthy weight, overweight, or obesity. It was hypothesized that the reduction in physical activity levels with advancing age increases with the increase in body weight status.

## 2. Materials and Methods

### 2.1. Study Population

This study is a secondary analysis of the 2016-2017 National Survey of Children's Health (NSCH). The NSCH is designed to produce national- and state-level data on the physical and emotional health of American children 0-17 years old [22]. The NSCH is sponsored by the US Department of Health and Human Services, Health Resources and Services Administration, and Maternal and Child Health Bureau and is conducted by the US Census Bureau. The 2016-2017 NSCH questionnaires were completed by mail and online. One child 0–17 years of age was randomly selected for detailed interview in each eligible household that contained age-eligible children. The survey respondent was the parent or guardian most knowledgeable about the child's health and health care. Details for the sampling methods and survey design were provided by NSCH [22, 23].

The present study used data from the 2016-17 NSCH Combined Data Set [24]. A total of 71,811 surveys were completed for 2016 and 2017 combined. 50,212 surveys were completed in 2016 and 21,599 in 2017, and the overall weighted response rate was 40.7% for 2016 and 37.4% for 2017 [23]. Data files, methodological reports, and other data-user resources were available online: https://www.childhealthdata.org. In the present study, we included all children aged 10 to 17 years with available information on BMI, and children with BMI less than the 5th percentile (underweight) were excluded.

### 2.2. Ethical Statement

The NSCH data and the permission for its use were obtained through a request from the Data Resource Center for Child and Adolescent Health (https://www.childhealthdata.org/dataset). More details regarding NSCH data availability and ethical standards are available at https://www.childhealthdata.org.

### 2.3. Variables

The primary outcome of this study was physical activity participation, which was measured by parent answer to the question, “During the past week, on how many days did this child exercise, play a sport, or participate in physical activity for at least 60 minutes?” [25]. The NSCH reported individual response to this question in four categories: 0 day, 1-3 days, 4-6 days, and all 7 days. This category was applied in our analysis for the descriptive statistics. Besides, dichotomous categories were also used for the descriptive statistics and binominal logistic regression analyses.

The independent variables were the child's age and weight status, as categorized from BMI percentiles. The NSCH reported the child's age in years. The weight status of children and teenagers are defined according to BMI percentile for age and sex [25]. In the NSCH, BMI were calculated based on parents' recollection of the selected child's height and weight and were categorized as less than the 5th percentile, 5th to 84th percentile, 85th to 94th percentile, and 95th percentile or above. Since previous studies have revealed that parents tend to overestimate height and underestimate weight of children younger than 10 years of age [26], the NSCH only reported BMI for children 10-17 years of age. In the present study, children with BMI less than the 5th percentile (underweight) were excluded. So the BMI in this study were categorized into three groups: BMI5th < 85th percentile (healthy weight), BMI85th < 95th percentile (overweight), and BMI ≥ 95th percentile (obese).

When describing the age distribution within different BMI groups, age was dichotomized as 10-11 years old and 12-17 years old (adolescents) according to the categories applied in the NSCH dataset. Covariates included sex (male/female), race (Hispanic/non-Hispanic white/non-Hispanic black/other or mixed race), highest education of adult in household (less than high school/high school or GED/some college or technical school/college degree or higher), household poverty status (0-199%/200-299%/300-399%/≥400%), current health insurance status (insured/not insured), and household tobacco use (no/yes, not inside the house/yes, inside the house). Covariates were selected from household environment and socioeconomic factors which were identified in previous publications as associated with childhood obesity and/or physical activity [13, 27–29].

### 2.4. Statistical Analysis

All statistical analyses accounted for the sampling design of NSCH by using survey weights, strata, and primary sampling units provided by NSCH [25]. Weighted population-based prevalence/percentages and 95% CI for selected characteristics and outcomes were estimated. Between-group comparisons were conducted using Wald chi-square test. Bonferroni correction was applied for multiple comparisons (the alpha value for each individual comparison equal to *α*/number of comparisons). Multivariate survey logistic regression analysis was used to detect associations between age, BMI category, and physical activity participation. Regression models were constructed for each of the two outcome measures, 0 day and <4 days, according to days of physical activity participation during the past 7 days. The independent variables were the child's age and BMI category. All models were adjusted for sex, race, highest education of adult in household, household poverty status, current health insurance status, and household tobacco use. Subgroup analyses were then conducted to investigate associations between age of years and days of physical activity participation by BMI percentile groups. All statistical analyses were performed using the SURVEY procedures in SAS v. 9.4 (SAS Institute, Cary, North Carolina). All tests of the hypothesis were two tailed with a type 1 error rate fixed at 5%.

## 3. Results

Data were analyzed on 33,056 US children age 10-17 years and BMI ≥ 5th percentile. Of the children included in the analysis, 71.13% were categorized into the BMI5th < 85th group, 15.15% were categorized into the BMI85th < 95th group, and 13.72% were categorized into the BMI ≥ 95th group.

The demographic characteristics and physical activity by active days during the 7 days before the survey are presented in Table 1. Significant differences were observed between three BMI groups on sex, highest education of adult in household, household poverty status, and physically active days (*p* < 0.017, Table 1). The distribution of age, race, and household tobacco use in the BMI85th < 95th group and the BMI ≥ 95th group was significantly different from that in the BMI5th < 85th group (*p* < 0.017), but the differences between the BMI85th < 95th group and the BMI ≥ 95th group were nonsignificant (*p* > 0.017).

Overall, 10.9% children had not been physically active for a total of at least 60 minutes on at least 1 day out of 7 days (0/7 days), and 19.4% children had been active all 7 days (7/7 days) (Table 1). The BMI5th < 85th group had the highest percentage of been active all 7 days (21.3%), while the percentages in the BMI85th < 95th group and BMI ≥ 95th group were significantly lower (16.4% and 14.8%, respectively, both *p* < 0.017) (Table 1).

**Figure 1:**
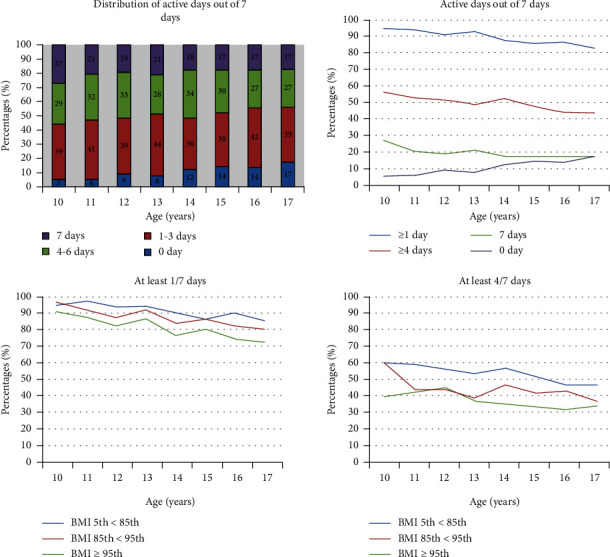
Distribution and weighted percentages of active days out of 7 days by age and BMI groups (2016-2017 National Survey of Children's Health). Active days were categorized as accumulating at least 60 minutes of physical activity at least one day and at least 4 days.

The distribution of percentages of active days by age is shown in Figure 1. The percentages of been physically active 0/7 days were ranged from 5% among 10-year-old children to 17% among 17-year-old children, while the percentages of been active 7/7 days declined from 27% among 10-year-olds to 17% among 17-year-olds. Figure 1 also shows the percentages of active days by active categories (≥1/7 days and ≥4/7 days), age, and BMI groups. The age-associated decline was observed for active ≥1/7 days in all BMI groups, while the percentages of been active ≥4/7 days fluctuated at some ages.

Table 2 shows the logistic regression results for associations between BMI, age, and physical activity. Children within higher BMI categories or at an older age tended to be less active. Compared with children at the age of 10 years, the odds of been active 0/7 days gradually increased with age (ORs ranged from 1.58 to 4.03 for significant associations). Similar trends had been observed between age and been active <4/7 days.

Table 3 shows the associations between age and physical activity by BMI subgroups. For all BMI subgroups, significant associations were observed between older ages and been active 0/7 days, although with different effect sizes. Using the age of 10 years as the reference group, the ORs of been active 0/7 days for age of 17 years in BMI5th < 85th, 85th < 95th, and ≥95th groups were 3.55, 7.48, and 3.84, respectively (all *p* values < 0.05). Age was also found to be significantly associated with been active <4/7 days in BMI5th < 85th and 85th < 95th groups (ORs ranged from 1.39 to 2.48 for significant associations). However, in the BMI ≥ 95th group, the associations between age and been active <4/7 days were not significant (*p* < 0.05).

## 4. Discussion

Regarding the topic of this study, there are three already known facts: (1) Most children do not meet the global and national physical activity recommendation [9, 10]. (2) Physical activity levels in children with overweight and obesity were significantly lower than their healthy-weight contemporaries [30–32]. (3) Physical activity levels in children decline with age [12, 13, 33]. And our findings from the 2016-2017 NSCH data were consistent with previous findings on those three aspects. It is noteworthy that our analysis showed that only 19.4% children 10-17 years of age had met the physical activity recommendation for children to accumulate a minimum of 60 minutes of physical activity each day. Moreover, 10.9% children had not been physically active on at least one day during the week. Among children within BMI ≥ 95th percentile, the percentage of been active 0/7 days was as high as 18.2%.

As the weight status and age are both inversely associated with physical activity participation, we hypothesized that the reduction in physical activity levels with advancing age increases with the increase in body weight status. Contrary to the hypothesis, our results showed that the association was strongest in the BMI85th < 95th group, while in the BMI ≥ 95th group, the active days were less associated with age. In other words, our investigation revealed that children with obesity tend to maintain high levels of inactivity throughout adolescence, while the physical activity participation in children with overweight is strongly and inversely associated with age. These findings highlight the importance of launching effective interventions targeting children with overweight and obesity to prevent the age-related decline in physical activity and to prevent the progression from overweight to obesity. Also, a question rises as to why the patterns of association between physical activity and age differed by weight status. Further studies are needed to investigate the reasons for this difference, which could help identify determinants of change in physical activity in children with different weight status.

Among the two outcome measures for active days out of 7 days (0/7 days and <4/7 days), the strongest associations with age were found for been active 0/7 days. From age 10 to 17 years, the percentages of been active 0/7 days gradually increased from 5% to 17%. And the percentage of been active 0/7 days in the BMI ≥ 95th group was even higher (18.2%). Significant associations were observed between ages and been active 0/7 days for all BMI groups (ORs range from 2.31 to 7.48). As there is a dose-response relation between physical activity and health status [3], the high prevalence of inactivity and the age-related decline in physical activity are serious challenges to public health. Our findings put emphasis on the necessity for the development of interventions that are designed for children with especially low levels of physical activity.

It has long been realized that it is difficult to increase physical activity among children with overweight and obesity, possibly because those children are vulnerable to body-related, resource, and social barriers to physical activity [19, 20, 34]. Result from a meta-analysis showed that interventions promoting physical activity had no effect on total physical activity among children with overweight and obesity, neither directly postintervention nor at long-term follow-up [19]. As there is no evidence for currently available interventions which are able to increase physical activity among children with overweight or obesity, new intervention strategies are needed [19]. Universal interventions targeting all children may have limited effects on promoting physical activity, because children with different health status and exercise habits may react differently to one intervention. Our findings on the associations between age and physical activity participation among children with healthy weight, overweight, and obesity demonstrate the importance for intervention programs to be tailored for children at different ages and with different weight statuses.

This study has several limitations. First, all data from the NSCH are provided based on parental report. The self-report of height, weight, and physical activity could have been subject to measurement bias and recall bias. Because parents tend to overestimate height and underestimate weight of children younger than 10 years of age [26], the NSCH only reported BMI for children 10-17 years of age. Although not entirely accurate, a study by Goodman et al. showed that self-reported height and weight can correctly classify 96% as to weight status [35]. Besides, in NSCH, the physical activity participation was reported as categorized data; therefore, we had not conducted qualitative analyses on active days. As the data was parent reported, parents may be unable to accurately quantify the physical activity of the child, and choosing a category would be more feasible than reporting an absolute number of active days. Another limitation is that the data were cross-sectional, so we could not analyze the changes of physical activity with age on the individual level. Longitudinal studies are needed to identify the individual trajectory of declines in physical activity and to identify factors associated with changes in psychical activity.

## 5. Conclusions

Physical activity participation was inversely associated with age among children with healthy weight, overweight, or obesity. The association was strongest among children with overweight and weakest among children with obesity. The identified patterns of association should be taken into account during the designing of intervention programs.

## Figures and Tables

**Table 1:** Weighted percentages and 95% CI for selected characteristics of children aged 10-17 years (2016-2017 NSCH).

Characteristics	Overall sample	BMI percentile groups					
		5^th^ < 85^th^ percentile	85^th^ < 95^th^ percentile		≥95^th^ percentile		
	*N* = 33056	*n* = 23513	*n* = 5007		*n* = 4536		
	% (95% CI)^a^	% (95% CI)^a^	% (95% CI)^a^	*p* ^b^	% (95% CI)^a^	*p* ^b^	*p* ^c^
Age (years)							
10-11	23.4 (22.3, 24.6)	21.6 (20.3, 22.9)	25.6 (22.8, 28.5)	**0.0003**	28.5 (24.9, 32.1)	**0.0005**	0.2265
12-17	76.6 (75.4, 77.7)	78.4 (77.1, 79.7)	74.4 (71.5, 77.2)		71.5 (67.9, 75.1)		
Sex							
Male	50.6 (49.3, 51.9)	49 (47.4, 50.5)	49.1 (45.7, 52.5)	**<0.0001**	58.4 (54.9, 62)	**<0.0001**	**0.0002**
Female	49.4 (48.1, 50.7)	51 (49.5, 52.6)	50.9 (47.5, 54.3)		41.6 (38, 45.1)		
Race							
Hispanic	24.4 (23, 25.8)	21.6 (19.9, 23.3)	28.6 (24.9, 32.3)	**<0.0001**	31.7 (27.7, 35.7)	**<0.0001**	0.0260
Non-Hispanic white	52.3 (50.9, 53.6)	56.6 (55.1, 58.2)	45.3 (42.2, 48.5)		41.4 (38.2, 44.6)		
Non-Hispanic black	13.9 (12.8, 14.9)	12 (10.9, 13)	15.9 (13, 18.8)		19.5 (16.3, 22.7)		
Other/mixed race	9.5 (8.9, 10.1)	9.8 (9.1, 10.6)	10.2 (8.6, 11.8)		7.4 (5.7, 9.1)		
Highest education of adult in household							
Less than high school	10.1 (8.8, 11.3)	8.1 (6.8, 9.5)	11.9 (8.5, 15.2)	**<0.0001**	16.1 (12.5, 19.7)	**<0.0001**	**<0.0001**
High school or GED	20.4 (19.2, 21.6)	17.9 (16.6, 19.3)	22.7 (19.7, 25.7)		28.1 (24.6, 31.5)		
Some college or technical school	23.5 (22.5, 24.6)	22 (20.8, 23.2)	25.9 (23, 28.7)		27.5 (24.2, 30.7)		
College degree or higher	46 (44.7, 47.2)	51.9 (50.4, 53.5)	39.6 (36.6, 42.6)		28.3 (25.7, 30.9)		
Household poverty status							
0-199%	40.3 (38.9, 41.7)	36.3 (34.6, 37.9)	43.1 (39.7, 46.6)	**<0.0001**	53.5 (49.9, 57.1)	**<0.0001**	**<0.0001**
200-299%	15.2 (14.2, 16.1)	14.4 (13.5, 15.4)	17.1 (14.4, 19.9)		16.2 (13.3, 19.1)		
300-399%	12.2 (11.4, 13)	12.8 (11.9, 13.7)	11 (8.8, 13.3)		11.1 (9.1, 13.2)		
≥400%	32.3 (31.3, 33.4)	36.5 (35.2, 37.8)	28.7 (26.2, 31.3)		19.1 (17.1, 21.2)		
Current health insurance status							
Insured	93.3 (92.5, 94.2)	93.8 (92.7, 94.9)	92.2 (89.7, 94.7)	0.3972	92.6 (90.5, 94.8)	0.3524	0.7975
Not insured	6.7 (5.8, 7.5)	6.2 (5.1, 7.3)	7.8 (5.3, 10.3)		7.4 (5.2, 9.5)		
Household tobacco use							
No	84.3 (83.3, 85.2)	85.7 (84.5, 86.8)	83.6 (81.5, 85.7)	**<0.0001**	79.2 (76.8, 81.6)	**<0.0001**	0.0215
Yes, not inside the house	12.8 (12, 13.6)	12 (10.9, 13)	13 (11.2, 14.8)		15.9 (13.8, 18)		
Yes, inside the house	3 (2.5, 3.4)	2.4 (1.9, 2.8)	3.4 (2.3, 4.5)		4.9 (3.7, 6)		
Physically active days out of 7 days^d^							
0 day	10.9 (10.1, 11.7)	8.9 (8.1, 9.7)	11.5 (9.6, 13.4)	**<0.0001**	18.2 (15.3, 21)	**<0.0001**	**0.0006**
1-3 days	39.6 (38.3, 40.9)	37.4 (35.9, 38.9)	43.8 (40.4, 47.2)		44.4 (40.8, 48)		
4-6 days	30 (28.9, 31.2)	32.3 (30.9, 33.7)	28.3 (25.2, 31.3)		22.7 (19.2, 26.1)		
Everyday	19.4 (18.3, 20.5)	21.3 (20, 22.7)	16.4 (13.8, 19.1)		14.8 (12, 17.6)		

BMI: body mass index; NSCH: National Survey of Children's Health. Values in bold are statistically significant (*p* < 0.017). *α* set at 0.017 for multiple comparisons (Bonferroni correction, *α* = 0.05/3). ^a^Weighted estimates of percentages and 95% CI. ^b^Compared with 5^th^ < 85^th^ percentile, using Wald chi-square test. ^c^Compared with 85^th^ < 95^th^ percentile, using Wald chi-square test. ^d^An active day was defined as the child had participated in physical activity for at least 60 minutes in that day.

**Table 2:** Associations between BMI, age, and physically active days (2016-2017 NSCH).

Factors	Active days out of 7 days^a^			
	0 day		<4 days	
	OR (95% CI)^b^	*p*	OR (95% CI)^b^	*p*
BMI groups				
5^th^ < 85^th^ percentile	1 (reference)		1 (reference)	
85^th^ < 95^th^ percentile	**1.37 (1.1, 1.71)**	**0.0058**	**1.46 (1.25, 1.7)**	**<0.0001**
≥95^th^ percentile	**2.12 (1.71, 2.63)**	**<0.0001**	**1.95 (1.62, 2.34)**	**<0.0001**
Age (years)				
10	1 (reference)		1 (reference)	
11	1.17 (0.69, 1.97)	0.5612	1.12 (0.88, 1.42)	0.3483
12	**1.93 (1.24, 2.99)**	**0.0033**	1.23 (0.98, 1.52)	0.069
13	**1.58 (1.03, 2.42)**	**0.0354**	**1.33 (1.06, 1.68)**	**0.0134**
14	**2.6 (1.71, 3.96)**	**<0.0001**	1.18 (0.95, 1.48)	0.1396
15	**3.23 (2.13, 4.87)**	**<0.0001**	**1.45 (1.17, 1.79)**	**0.0006**
16	**3.06 (2.04, 4.6)**	**<0.0001**	**1.64 (1.3, 2.07)**	**<0.0001**
17	**4.03 (2.71, 6)**	**<0.0001**	**1.68 (1.36, 2.08)**	**<0.0001**

BMI: body mass index; NSCH: National Survey of Children's Health. Values in bold are statistically significant (*p* < 0.05). ^a^An active day was defined as the child had participated in physical activity for at least 60 minutes in that day. ^b^Survey logistic regression results adjusting for sex, race, highest adult education, household poverty status, current health insurance status, and household tobacco use.

**Table 3:** Subgroup analysis for associations between age and physically active days by BMI categories.

BMI subgroups	Age (years)	Active days out of 7 days^a^			
		0 day		<4 days	
		OR (95% CI)^b^	*p*	OR (95% CI)^b^	*p*
5^th^ < 85^th^ percentile	10	1 (reference)		1 (reference)	
	11	0.9 (0.49, 1.67)	0.74	1.01 (0.76, 1.34)	0.9274
	12	1.45 (0.83, 2.55)	0.1914	1.2 (0.92, 1.56)	0.1754
	13	1.47 (0.88, 2.46)	0.1433	1.23 (0.93, 1.61)	0.1444
	14	**2.26 (1.39, 3.67)**	**0.001**	1.12 (0.86, 1.44)	0.4013
	15	**3.08 (1.9, 5)**	**<0.0001**	**1.39 (1.09, 1.77)**	**0.0083**
	16	**2.44 (1.55, 3.85)**	**0.0001**	**1.65 (1.28, 2.12)**	**0.0001**
	17	**3.55 (2.23, 5.65)**	**<0.0001**	**1.65 (1.29, 2.1)**	**0.0001**
85^th^ < 95^th^ percentile	10	1 (reference)		1 (reference)	
	11	1.8 (0.73, 4.43)	0.1991	**1.9 (1.14, 3.17)**	**0.0135**
	12	**3.49 (1.56, 7.83)**	**0.0024**	**1.85 (1.12, 3.03)**	**0.0154**
	13	**2.31 (1.1, 4.84)**	**0.0265**	**2.17 (1.25, 3.76)**	**0.0058**
	14	**4.76 (2.08, 10.89)**	**0.0002**	1.69 (0.92, 3.1)	0.091
	15	**4.87 (2.38, 10)**	**<0.0001**	**2.03 (1.23, 3.35)**	**0.0055**
	16	**5.68 (2.57, 12.55)**	**<0.0001**	**1.86 (1.06, 3.28)**	**0.0313**
	17	**7.48 (3.6, 15.52)**	**<0.0001**	**2.48 (1.47, 4.17)**	**0.0007**
≥95^th^ percentile	10	1 (reference)		1 (reference)	
	11	1.39 (0.46, 4.21)	0.5642	0.97 (0.51, 1.81)	0.913
	12	2.25 (0.9, 5.64)	0.083	0.88 (0.5, 1.54)	0.6536
	13	1.51 (0.59, 3.86)	0.3893	1.16 (0.65, 2.08)	0.6195
	14	2.36 (0.92, 6.09)	0.0753	1.11 (0.66, 1.86)	0.695
	15	2.58 (1, 6.7)	0.0508	1.25 (0.69, 2.26)	0.4634
	16	**3.42 (1.35, 8.67)**	**0.0096**	1.4 (0.7, 2.83)	0.345
	17	**3.84 (1.59, 9.26)**	**0.0027**	1.24 (0.71, 2.17)	0.4532

BMI: body mass index; NSCH: National Survey of Children's Health. Values in bold are statistically significant (*p* < 0.05). ^a^An active day was defined as the child had participated in physical activity for at least 60 minutes in that day. ^b^Survey logistic regression results adjusting for sex, race, highest adult education, household poverty status, current health insurance status, and household tobacco use.

## Data Availability

The NSCH data and the permission for its use were obtained from a request from the Data Resource Center for Child and Adolescent Health (https://www.childhealthdata.org/dataset). More details regarding NSCH data availability and ethical standards are available at https://www.childhealthdata.org.
